# Perspectives for plant biology in space and analogue environments

**DOI:** 10.1038/s41526-023-00315-x

**Published:** 2023-08-21

**Authors:** Veronica De Micco, Giovanna Aronne, Nicol Caplin, Eugénie Carnero-Diaz, Raúl Herranz, Nele Horemans, Valérie Legué, F. Javier Medina, Veronica Pereda-Loth, Mona Schiefloe, Sara De Francesco, Luigi Gennaro Izzo, Isabel Le Disquet, Ann- Iren Kittang Jost

**Affiliations:** 1 Department of Agricultural Sciences, University of Naples Federico II, via Università 100, 80055 Portici (NA), Italy; 2 SciSpacE Team, Directorate of Human and Robotic Exploration Programmes, European Space Agency (ESA), Noordwijk, Netherlands; 3 Institute of Systematic, Evolution, Biodiversity, Sorbonne University, National Museum of Natural History, CNRS, EPHE, UA, 45, rue Buffon CP50, 75005 Paris, France; 4 Centro de Investigaciones Biológicas Margarita Salas - CSIC, Ramiro de Maeztu 9, 28040 Madrid, Spain; 5 Belgian Nuclear Research Centre (SCK CEN), Biosphere Impact Studies (BIS), Boeretang 200, 2400 Mol, Belgium; 6 Université Clermont Auvergne, INRAE, PIAF, F-63000 Clermont-Ferrand, France; 7 GSBMS/ Evolsan UFR Santé, University of Toulouse III, Toulouse, France; 8 NTNU Social Research, Centre for Interdisciplinary Research in Space (CIRiS) Dragvoll Allé 38 B, 7049 Trondheim, Norway

**Keywords:** Plant sciences, Cell biology

## Abstract

Advancements in plant space biology are required for the realization of human space exploration missions, where the re-supply of resources from Earth is not feasible. Until a few decades ago, space life science was focused on the impact of the space environment on the human body. More recently, the interest in plant space biology has increased because plants are key organisms in Bioregenerative Life Support Systems (BLSS) for the regeneration of resources and fresh food production. Moreover, plants play an important role in psychological support for astronauts. The definition of cultivation requirements for the design, realization, and successful operation of BLSS must consider the effects of space factors on plants. Altered gravitational fields and radiation exposure are the main space factors inducing changes in gene expression, cell proliferation and differentiation, signalling and physiological processes with possible consequences on tissue organization and organogenesis, thus on the whole plant functioning. Interestingly, the changes at the cellular and molecular levels do not always result in organismic or developmental changes. This apparent paradox is a current research challenge. In this paper, the main findings of gravity- and radiation-related research on higher plants are summarized, highlighting the knowledge gaps that are still necessary to fill. Existing experimental facilities to simulate the effect of space factors, as well as requirements for future facilities for possible experiments to achieve fundamental biology goals are considered. Finally, the need for making synergies among disciplines and for establishing global standard operating procedures for analyses and data collection in space experiments is highlighted.

## Introduction

At the beginning of space exploration, research in space life science was focused on the human body to gain a fundamental understanding on the responses to the stressful space environment, to ultimately prevent health risks and protect astronauts by managing space-induced pathological issues^1,2^. In the last decades, increasing interest has been raised towards plant space biology due to the awareness that cultivation of higher plants in space is a requirement for long-duration human missions, where the regeneration of resources and plant-based food production onboard must be increased at the expense of re-supply from Earth^3^.

Plants play a crucial role in Bioregenerative Life Support Systems (BLSS). Artificial ecosystems such as the BLSS are high-technology systems, based on the integration of physico-chemical and biological processes, to support long interplanetary missions^4,5^. The BLSS concept includes several interconnected compartments in which different organisms are used to sequentially recycle resources^6,7^. Within a BLSS, the photoautotrophic compartment enables the production of edible biomass, oxygen, and water as resources for the astronaut, starting from carbon dioxide, wastewater and other wastes. An example of BLSS is the MELiSSA (Micro-Ecological Life Support System Alternative) loop by the European Space Agency (ESA) that aims to sustain astronaut life in space missions to reduce the initial payload and dependency from Earth^8^. The MELiSSA loop is made of interconnected compartments among which the requirements of the higher plant compartment need to be fulfilled at least in part by the outputs of other compartments^9^. As well, the species/cultivar choice and environmental control are dictated by the need to meet the requirements of the other compartments (e.g., oxygen requirements of the animal crew) in addition to the generally accepted requirements including short cultivation cycles, reduced plant size, high harvest index, and resistance to diseases. Increasing effort is posed on plants to produce healthy food specifically designed to sustain crew in long term missions^3,10,11^.

Additionally, research in space-plant biology and space-agriculture is required alongside the strong efforts to increase the knowledge on human biology in space with the aim to predict risk factors, prevent disease(s), and implement effective countermeasures to manage health emergencies during missions. Among countermeasures for assuring astronauts' well-being against degenerative diseases and psychological issues, there is the introduction, in their nutrition, of plant-derived fresh food produced directly on board or within a habitat. Further, there may also be benefits for crew mental health by allowing crews to participate in plant cultivation in the living and/or working pressurized modules in space^12,13^.

## What are the main constraints to plant cultivation in space?

From an applied point-of-view, the design and realization of BLSS require a better understanding of plant acclimation and adaptation processes that determine the plant’s capability to complete a full life cycle in space (producing viable offspring) for both model plants and crops. As a matter of fact, the altered growth and behaviour of plants in space can alter the input/output balance between the different compartments as well as the potential nutritional value of the derived fresh-food. Indeed, as any other living organism, plants’ survival and reproduction strongly depend on interactions with environmental factors (e.g. temperature, light, oxygen/carbon dioxide, water, and volume availability) including the novel space factors (e.g., altered gravity and ionizing radiation). Among different environmental factors, altered gravity and ionizing radiation are recognized to be the main constraints for growth in space conditions. In the following section, we briefly summarize the current knowledge about the effects of microgravity and radiation on plants.

## What is already known about the effects of microgravity and radiation

To date, European scientists studying plant space biology have focused on understanding the effects of altered gravity on several plant systems (e.g. different species/cultivars, target organs/tissues). Recently, more attention has been dedicated to the effect of ionizing radiation (IR) so far as it represents a clear challenge for exploratory-class missions^14^. Altered gravity and exposure to IR can induce changes in gene expression, cell proliferation and differentiation, signalling and physiological processes (see precise data and references in the following paragraphs). The expected consequence of these changes would be serious structural and functional alterations at the whole organism level. In addition, since these cellular and molecular changes are detected in early developmental stages, developmental alterations would also be expected. However, altered tissue organization, organogenesis and functioning have not always been detected under spaceflight conditions. For example, none of these effects has been reported to occur when plants have been cultured in space: adult organisms were produced with no evident aberrations and the entire *seed-to-seed* life cycle of plants was successfully achieved under spaceflight conditions^15,16^. Interestingly, this phenomenon is not exclusive to plants. It was first detected and reported by Marco et al. ^17^ in the fruitfly *Drosophila melanogaster* and defined as “an apparent paradox”. Up to now, this paradox has not been resolved and remains an important challenge for space biology research.

### Effect of microgravity and altered gravity on plant growth and reproduction

Since 475 million years ago, land plants have evolved under the same 1 *g* gravity level. Early pioneering space experiments have demonstrated that plants are able to survive and grow in space, although morpho-physiological alterations were soon reported in crop species as lettuce and lentil^18,19^. In early research, deficiencies in the experimental setup and in used facilities were sometimes responsible for confusing and contradictory results. For example, nutrient absorption has been reported to increase, decrease or remain unchanged under reduced gravity, likely due to hardware limitations and species-specific responses^20^. Now, however, there is evidence that microgravity in space environment does not prevent plant growth and reproduction but causes serious alterations in plant physiology and development^21^. Plants respond to changes in mechanical inputs, therefore gravity-related research offers a unique opportunity to elucidate the mechanisms of gravity perception whose knowledge is necessary to better understand fundamental processes and the physiological changes in space. Moreover, in exploratory-class long-term missions, plants will face not only a lack of gravity (or residual gravity) during spaceflight (and inside the International Space Station - ISS) but also the lunar gravity (0.17 *g*) and the Martian gravity (0.37 *g*) which are substantially lower to what plants normally experience on Earth. Indeed, in forecasting possible acclimation of plants in environments with altered gravity, thresholds for gravity sensing should be considered. Previous studies indicated that the threshold acceleration perceived by lentil roots in spaceflight can be in the order of 10^–3^ *g* or lower^22^, but the effects of altered gravity on cell cycle have been detected at higher levels, intermediate between Moon and Mars gravity, in simulation experiments on Earth^23^. Similarly, the range for the attenuation of phototropism in higher plants occurs in the same range 0.1–0.3 *g*^24^.

#### Plant developmental patterns and gravity perception: from gene expression to cell cycle and organ development

Most studies to reveal the effect of altered gravity on plant development have been focused on the root meristem^25^. Experiments under microgravity simulation and onboard the ISS revealed the disruption of the meristematic competence in seedlings, i.e. the loss of the coordinated progress of cell proliferation and cell growth that characterizes meristematic cells under gravity conditions on Earth^26–28^.

An acceleration in the cell cycle was detected in in-vitro cultured *Arabidopsis* MM2d cells grown in simulated microgravity conditions produced in a Random Positioning Machine (RPM), resulting from downregulation of genes involved in the G2/M transition checkpoint and upregulation of genes controlling the G1/S transition. Other phenomena were the downregulation of significant genes for ribosome biogenesis and the corresponding depletion of the levels of nucleolar proteins, the depletion of the nuclear transcription and an increase in chromatin condensation, related to the epigenetic regulation of gene expression^29,30^. Experiments using different levels of reduced gravity and hypergravity indicated modulation of alterations for each level, with Mars gravity inducing milder alterations^23,31^. Indeed, studies on gravitropism and phototropism have shown that the reduced gravity level on Mars of 0.38 *g* should not be a major problem for plant growth^24^.

Auxin is a key factor regulating the connection between perceived stimuli and cellular responses, controlling the balance between cell proliferation and cell differentiation, regulating the cell cycle progression and the coordination between cell growth and cell division^32–35^. The role of the LAZY proteins in gravity sensing has been recently described, being crucial players linking gravity perception and gravitropic curvature through the proper redistribution of auxin, the relocation of the auxin efflux carrier PIN-FORMED (PIN) proteins for the tropic response of both roots and shoots^36^. However, the role of auxin and its polar transport in plant growth and development under microgravity conditions is not fully understood and requires further investigation to solve controversial concerns due in part to its complex interaction with cytokinin. For example, it has been shown in *Arabidopsis* that real microgravity does not influence the distribution of auxin in the primary root, whereas it affects that of cytokinin^37^. In contrast, the inhibition of pea hypocotyl growth in microgravity is correlated with an attenuation of polarized auxin transport, a decrease in auxin levels and an increase in cytokinin levels^38^.

Parallel transcriptomic experiments exploring how simulated (i.e. clinorotation, RPM and diamagnetic levitation) and real microgravity change gene and protein expression have shown a complex response of plants at early developmental stages (mostly *Arabidopsis* seedlings), involving reprogramming of the gene expression pattern^39^. Specific genes of response to gravity alteration have not been found, while the main and most frequent targets of this gene reprogramming are: genes coding for heat shock-related elements, cell wall remodelling factors, oxidative burst intermediates and components of the general mechanisms of plant defence against stressors, being differently affected at the different spaceflight, lunar and martian *g* levels^40^. The results of several experiments in space using a centrifuge to produce different gravity levels showed a differential response to each level, triggering different adaptive responses, involving changes in the regulation of different sets of genes. Changes in gene expression were lower under Mars gravity compared to microgravity on the ISS^40,41^.

#### Interaction with other factors

Many plant responses are primed by the interaction between gravity and other physical, chemical, or biological factors. In the last decades, orbital platforms enabled comprehensive studies on the mechanisms underlying plant growth in microgravity^42^. Still, there is a need to further investigate the interactions between gravity and other environmental factors including temperature, light, oxygen/carbon dioxide, water availability, electric and magnetic fields, especially in the framework of plant morphogenesis and tropic responses, also considering intra-specific genotypic variability^43,44^. With the exception of a limited number of studies concerning microgravity interactions with magnetic fields, water or chemical stimuli on crops (i.e. flax, cucumber and carrot)^45–47^, previous research has mainly focused on the interactions between gravity and light. It has been shown that the sensitivity of plants to light is influenced by microgravity and that phototropic curvature of the shoot and root organs are largely affected by changes in gravity conditions^48^ Experiments performed using the EMCS onboard the ISS or the ESA ground-based facilities resulted in discovering novel phototropic responses of plants, both the model *Arabidopsis* and *Brassica oleracea*, proving that the interaction between gravity and light changes according to the magnitude of g-force^49–51^. In this framework, light quality has a prominent role in determining the direction and strength of phototropic responses of shoots and roots, with major differences between blue and red wavelengths under a wide range of gravity levels^51,52^. On the other hand, previous studies also showed that light can control the sensitivity of plants to gravity through phytochrome-regulated pathways, indicating that phytochromes play a key role in integrating multiple environmental stimuli^53^.

Gene expression studies with different levels of altered gravity showed that the adaptive response appeared enhanced by red light photostimulation. Red light activates cell proliferation and ribosome biogenesis in pea^54^, and in the “Seedling Growth” series of experiments in the ISS, red light caused a concerted upregulation of marker genes for cell proliferation, cell growth and auxin polar transport^55^. Experiments with different levels of gravity (microgravity, Mars gravity and ground control gravity) with and without red light photoactivation have shown that red light restored the auxin distribution patterns which appeared altered under microgravity, while in roots grown at 0.3 *g*, the auxin polar transport was slightly altered, irrespective of photoactivation^56^. The red light was also shown to counteract the decoupling between cell proliferation and growth in root meristems reported in earlier experiments^41^.

Gene expression alterations, evaluated by RNA-seq, showed different responses to different gravity levels and modulation of gene expression by red light photoactivation. As an example, Mars gravity level induced an adaptive response, consisting of the activation of environmental acclimation-related transcription factors (WRKY and NACs families), especially in photostimulated samples^41^.

It appears clear that plant cultivation in future space missions implies research-based strategies that involve gravity-substituting factors (e.g., light) to counteract the effects of microgravity or partial gravity conditions such as on the Moon and Mars. Nowadays, the technological advancement in cultivation systems is providing effective and affordable tools to control environmental factors for plant growth in space with the possibility of using external cues for both application and research purposes^42^. Gravity might be replaced by specific stimulation in terms of light wavelengths and photon flux density for the regulation of plant growth and development. Prospectively, other factors are expected to play such a role including water, electric and magnetic fields, chemicals, or microorganism, but further investigation is needed.

#### Seed-to-seed cycle

Nowadays, it is crucial to delve into the mechanisms by which altered gravity conditions can affect plant reproduction and seed viability, in order to develop cultivation strategies for the improvement of plant-based BLSS in future human settlements on the Moon and Mars^57^. The completion of the *seed-to-seed* cycle, in fact, will be essential to produce viable seeds to be used for the cultivation of plants over time without relying on terrestrial supply.

Early studies with plants grown for extended periods in microgravity reported an overall reduction of plant growth and difficulties in the transition to the reproductive stage^58^. Since the first seed production in space by *Arabidopsis thaliana* plants in 1982, a few experiments on plant reproduction have been performed^59^. Overall, previous studies showed that the *seed-to-seed* cycle can be accomplished in most species tested in microgravity, although with reduced quality of embryos and seeds produced by plants due to delayed embryo development, modification in storage reserves, delayed starch use in cotyledons, and decreased cell number in cotyledons^15,58,60–62^. Furthermore, experiments using simulated microgravity (e.g., clinorotation) showed significant alterations during the development of male gametes in several crop species^63^.

Given that most studies have investigated the effect of microgravity on early stages of plant development with sporadic studies on plant reproduction, there is a large gap of information to be filled to fully the effects on the growth of plants in the adult stage and on plant reproduction.

### Effects of ionizing radiation on plant growth and development

Outside Low Earth Orbit, IR is variable in space and time and can severely constrain organisms’ growth^3,14^. IR can cause direct damage to the structures encountered, but also indirect due to the generation of reactive oxygen species (ROS)^64,65^. The oxidative stress due to ROS production may damage important components of plant cells, including lipids and proteins, but especially DNA^66,67^. The degree of direct DNA damage and proper functioning of DNA repair systems determine the consequences of IR exposure for plants at morpho-structural and physiological level^68^.

Nevertheless, plants’ responses to IR are not fully understood yet. Experiments in space where plants, either the model *Arabidopsis* or crops such as beans and tomato, were exposed to cosmic radiation and on the ground with exposure to low- and high- linear energy transfer (LET) radiation have shown that IR can have positive, null or negative effects on plants, at genetic and morphophysiological levels depending on IR properties and plant intrinsic factors such as type of radiation, its LET, exposure time (acute or chronic), dose, plant species/cultivar, developmental stage at the time of irradiation^69^. The effect of IR is also tissue-specific and depends on tissue architecture: complex tissues in beans and tomatoes seem less sensitive to damage^70,71^, and, on the contrary, the meristematic cells are the most sensitive to radiation^72^.

High-LET radiation, like protons and heavy ions, is more harmful in inducing genetic mutations compared to low-LET radiation such as X- and γ-rays^73,74^. Concerning the dose of exposure, high doses (>100 Gy for seeds; >50–70 Gy for vegetative stages) can lead to harmful outcomes, such as reduced levels of photosynthesis and germination, embryo lethality, loss of apical dominance, dwarf architecture, altered leaf anatomy, and accelerated senescence^67,69,75^. Low doses of IR, on the other hand, appear to induce hormetic response in plants, stimulating germination, growth, photosynthetic and respiration rate, improving the content in chlorophyll, carotenoids, non-enzymatic (ascorbic acid, glutathione, and anthocyanin) and enzymatic antioxidants (ascorbate peroxidase, catalase, and superoxide dismutase) and phenolic compounds, effective in counteracting the oxidant action of ROS, thus increasing plant nutritional value and radioresistance^75–80^.

Most of the studies performed on plants have been conducted by irradiating dry seeds with acute doses (due to limitations in the volume and time of irradiation availability)^75,81^. Moreover, most of the studies involving crop species have mainly been focused on using IR to introduce genetic variation and selecting plant cultivars with specific traits^82^. Only a few studies have considered the effects on the yield, nutritional value as well as interaction with other factors. A recent study has indicated that the effect of X-rays delivered to germinated seeds at different doses is strongly influenced by light quality during subsequent cultivation^83^.

As a result, little information is available on the variation of radiosensitivity during the different phenological phases in the case of acute exposure and on the effects of chronic exposure. Indeed, it is important to emphasize that resistance to large doses of radiation delivered in an acute way (in shorter times than those necessary for the repair of cellular damage) often does not translate into resistance to chronic exposures for multiple generations and vice versa^84^. Therefore, in the sight of future space exploration, the time is ripe for increasing the efforts to investigate plant responses to chronic low dose-rate and high LET radiation to clarify all the processes and mechanisms behind the radioresistance phenomenon^14^.

## Knowledge gaps in microgravity and radiation research in plant biology

**Fig. 1:**
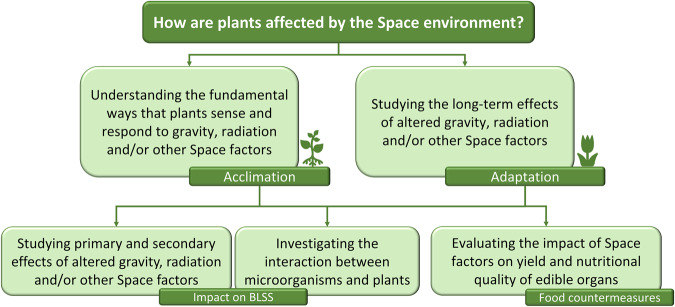
Schematic view of the main knowledge gaps in plant biology which regard understanding processes of acclimation and adaptation to space factors. Understanding such processes is fundamental to evaluate the impact on the functioning of BLSS and on the value of plant-derived food for the integration of astronauts’ nutrition.

According to the current scientific knowledge on plants’ responses to space factors, in order to successfully achieve the *in-progress* target of crewed missions in space, it is necessary to fill knowledge gaps in plant biology. They can be synthesized in the following five points (Fig. 1).

1 - Understanding the fundamental ways that plants sense and respond to gravity alone or in combination with radiation and other space and environmental/cultivation factors. This point is mainly related to short-term effects and acclimation strategies and includes studies on tropisms and morphogenesis.

2 - Studying the long-term effects of altered gravity, radiation and/or other space environment factors on plants and understanding how plants adapt to this new kind of environment.

3 - Studying primary and secondary effects of altered gravity, radiation and/or other space factors on plant growth and reproduction.

4 - Investigating the interaction between microorganisms and plants (beneficial and pathogenic) under space conditions in light of the realization of cultivation modules in BLSS.

5 - Investigating the effects of space factors on yield and nutritional value and quality (e.g. production of nutraceutical compounds) of edible organs targeting the use of plant-derived fresh food as countermeasures to improve astronauts’ health.

Points 3–5 can be investigated in the short-period, thus being mainly targeted to unravelling the acclimation strategies of plants to space factors. The same points can be investigated in the long-term and over multiple generations to evaluate the heritability of varied traits, hence leading to adaptation. In the latter case, constraints to reproduction become crucial to be analyzed.

Overall, the main objective of research activities in plant space biology is to reveal potential acclimation and adaptation mechanisms and processes in the response of plants (crops and model species) to microgravity, partial gravity and variable space radiation in combination with other environmental/cultivation conditions (e.g. airflow, light), through the developmental phases of a whole life cycle. This will allow the resolution of the “apparent paradox” between molecular and cellular effects *versus* organismic and developmental effects by understanding the mechanisms by which plants overcome the impacts occurring at early plant life stages after exposure to spaceflight conditions.

To understand if exposure to adverse space conditions potentially leads to acclimation and adaptation, the long-term responses of plants have to be investigated through sequential studies of plants after different times of exposure to single or multiple space factors, at different phases of the plant development. The acclimation has to be studied during the life cycle of the plant and adaptation has to be studied after several generations of plants exposed to a space environment.

Knowledge of plant-microorganism interactions is also important. Plants naturally attract microorganisms, some detrimental to plant health while others establish symbiotic relationships. Plants also have endophytes (bacteria and fungi) living between plant cells, some of which are transmitted to the following generations. Understanding the effect of the space environment on the relations between microorganisms and plants can help assess risks to future crew food supply or discover opportunities for microorganisms-mediated enhanced crop yield.

Unravelling the plant acclimation and adaptation processes, responsible for producing essentially viable adult individuals, is important not only for our fundamental understanding within plant biology but also for the realization of BLSS.

Indeed, addressing the five points mentioned above would allow to evaluate:

- How the growth processes and regeneration capacity of plants are affected by space factors and thus impact the cultivation requirements in producers’ modules of BLSS.
- If reproductive success is achieved and whether multiple generations of plants are possible to obtain, in order to guarantee the possibility to produce seeds for successive cultivation cycles.
- If and how the yield and nutritional value and quality of edible organs are affected and thus impact the astronauts’ nutrition.

The two first points regard every plant species, not only crops but also model species such as *Arabidopsis*, while the second and third mainly refer to crop species.

## Facilities in space and on Earth to study the effects of microgravity and radiation on plants

**Fig. 2:**
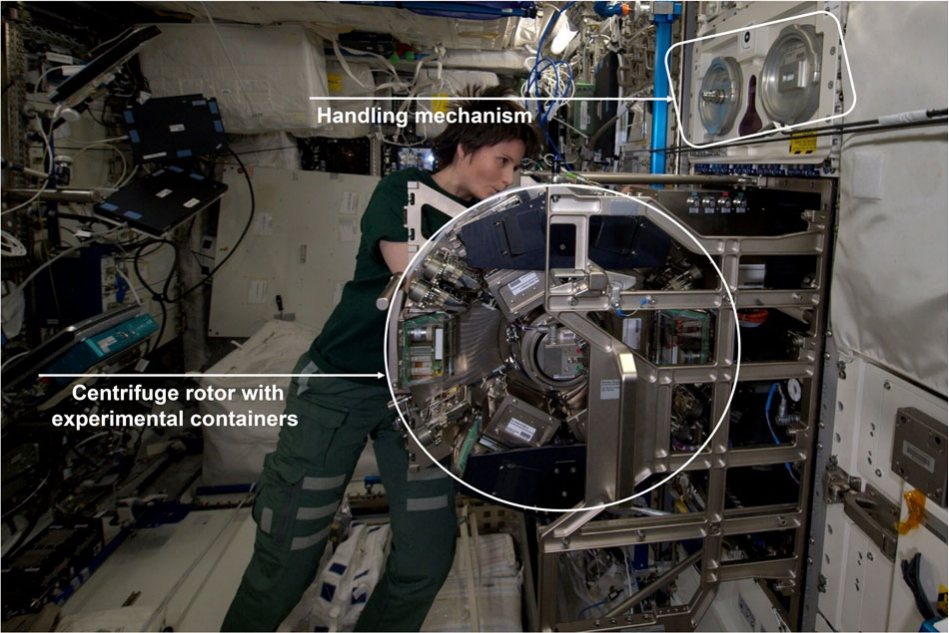
ESA astronaut Samantha Cristoforetti working with ESA’s Biolab facility in the Columbus laboratory on the International Space Station. It appears evident the presence of the centrifuge but the strict limitations of volume available for the experimental containers. Credits: ©ESA/NASA.

The ISS is an important research platform to study not only the effects of reduced gravity but also the long-term consequences of low dose space radiation on plants. The daily dose received in the ISS has been estimated at 0.5 mSv, assessed by physical dosimetry using phantoms^85^_,_ which is about 100 times higher than the dose on Earth, and about 2.6 and 1.28 times lower than the dose on the Moon and Mars surface^86,87^. By combining studies on ISS of plants exposed to various space factors (gravity/space radiation) and environmental cultivation conditions (e.g. different airflow or light conditions), additional required knowledge for future space agriculture can be obtained. Long-term experiments and full cycle studies of plants require a minimum cultivation area. Although the growth area has become larger in the newer ISS facilities (compared to systems such as Kubik and Icecubes), they are still considered too small for crops and full life cycles. When a 1 *g* or simulated Moon/Mars gravity exposure is required, the growth area is limited due to the diameter of the centrifuge rotor that has to fit into standard-sized racks on the ISS. An extensive review was done on the space plant growth systems where more than 20 systems are described^42^. The ESA BIOLAB facility on ISS has 4 Advanced Experiment Containers (AEC) on two rotors with a limited growth area per AEC (http://wsn.spaceflight.esa.int/docs/Factsheets/8%20Biolab%20LR.pdf) (Fig. 2) (Table 1). The BIOLAB allows unique experimental equipment to be built inside the AEC, and the available growth area for the plant will depend on the instrumentation required in the AEC to perform the experiment but still remains limited. The NASA systems such as Vegetable Production System (VEGGIE, 2014) and Advanced Plant Habitat (APH, 2017) have increased crop growth area, but they are still relatively small^88–90^. The APH is a closed, controlled growth system with full environmental monitoring and control. The VEGGIE system is simpler, with less control, and designed for more crew interaction (Table 1). For plant experiments where smaller volumes are required, the NASA Advanced Biological Research System (ABRS) is available with two experimental research chambers (growth area 0.053 m^2^). The chamber has light and environment control, and one of the two chambers is outfitted with Green Fluorescent Protein Imaging System. The JAXA Plant Experiment Unit (PEU), available on the Kibo laboratory on ISS, is equipped with a LED lighting system with red and blue LEDs, a growth chamber (growth area 0.027 m^2^), an automated watering system and a CCD camera.

**Table 1:** Comparison of key parameters for plant cultivation for ESA Biolab (http://wsn.spaceflight.esa.int/docs/Factsheets/8%20Biolab%20LR.pdf), NASA VEGGIE^88^, and NASA APH^86,88^.

Parameter	BIOLAB (ESA)	VEGGIE (NASA)	APH (NASA)
Growth area and volume	1. 6 cm × 6 cm × 10 cm 2. 10.8 cm × 15 cm × 13.7 cm	1. 47 cm × 1300 cm^2^ 2. 41.9 cm × 1300 cm^2^	1. 45 cm × 1708 cm^2^
Light provision	White adjustable 1.7–11.1 W/m2 PAR, Infra-Red	100–500 μmol m^−2^ s^−1^ Red, Blue, Green	0–1000 μmol m^−2^ s^−1^ White, Red, Blue, Green, Far-red
Temperature range	18–40 °C	ISS cabin equivalent	18–30 °C
Humidity control	Yes	No	Yes
Gas regulation	Yes	No	Yes
Trace gas removal	Yes	No	Yes
Centrifuges (fractional gravity)	2	No	No
Experiment positions	4–6 containers	1 container	4 root modules
Growth substrate/root support	Experiment dependent	Pillows with growth substrate, controlled-release fertiliser and seeds Water reservoir	2 moisture sensors O_2_ sensor Water/nutrient delivery system
Observation	Images (top view) Video	ISS camera used by crew	2 colour cameras (top and side view) Infrared camera

### What is further required?

Larger growth areas, where both model plants and crop plants can be cultivated for long-term experiments (during the whole life cycle), are needed to fully understand the plant acclimation and adaptation to the space conditions. Such a facility requires the control of environmental factors (including temperature, light, gasses, relative humidity, airflow, nutrient solution, and watering), and continuous imaging of the plant growth (e.g., visible light images, IR images using thermal cameras, fluorescence images). Environmental and imaging data should as a minimum be partially and periodically downloaded for feedback from the science teams collaborating from the ground. To collect data for the molecular, cell and anatomical studies, plant samples need to be harvested after sequential exposures to reduced gravity, preserved on ISS either by freezing or chemical fixation and brought back to the ground for analyses. In addition, the possibility to make microscopy analyses onboard has become a reality with the ESA/DLR Fluorescence Microscopy Analysis System (FLUMIAS), a high-resolution fluorescence microscope for live-cell imaging that is available on the ISS^91^.

A specific point affecting the technical needs of space facilities for plant culture is the possibility of performing comparative studies in spaceflight at different levels of gravity, including the Moon and Mars gravity, as well as the inflight 1 *g* control. The latter is of the highest importance and makes mandatory the implementation of centrifuges in the facilities used for plant cultivation in the ISS. The advanced sophisticated facilities for plant cultivation now available on ISS, such as VEGGIE, APH and the Exposed Roots On-Orbit Test System (X-ROOTS), which have proven successful in the cultivation of a wide range of plant species, are not equipped with centrifuges. ESA in 2018 decommissioned the European Modular Cultivation System (EMCS), a highly useful facility in which different successful experiments were carried out, some of them including pioneering comparative analyses at different levels of gravity in addition to the necessary in flight 1 *g* controls. Later on, in 2020, ESA promoted a discussion group to adapt the ESA Biolab facility to harbour experiments using crop species and encompassing the full plant life cycle while being exposed to different gravity levels. Whatever the final decision adopted, the need for an effective plant cultivation facility in space with the capabilities mentioned above, as a key to gaining knowledge to support human life in space exploration, is becoming more and more urgent within the next years.

## Applications and benefits for Earth

Understanding how plants are able to grow and adapt to space conditions will ensure reliable and predictable food supplies for human space exploration and has strong synergies with the United Nations Sustainable Development Goals, global food security and circular economy.

The sophisticated agro-technologies developed for space applications (e.g., innovative lighting, watering and nutrient delivery systems, fine environmental monitoring with automated control, imaging systems to analyze plant health, etc.) bring innovations to agriculture on Earth to improve sustainable plant cultivation and food production. For example, developing volume-saving, highly efficient plant growth controlled environments is beneficial for food and drug production particularly in densely populated urban areas in line with vertical farming technologies, in underground facilities with no natural light source, and in general in extreme environments such as deserts and poles.

Developing smart and safe pest control methods is applicable in confined volumes where aerosols are undesirable and natural predation for the reduction/removal of pests is not possible.

Besides these more human operational and exploration-oriented goals, it should also be stressed that the unique near weightlessness environment, as well as the high levels of ionizing radiation, also provide a research laboratory that cannot be obtained in on-ground laboratories and as such can answer specific and fundamental questions in life sciences. Improving the knowledge on how plants respond to ionizing radiation can provide information applicable in all the fields in which radiation is studied on Earth ranging from breeding programs, decontamination methods and radioecology.

## Future perspectives and recommendations in the short and long term

Today there is a need for fundamental research that goes beyond the demonstration of plants’ ability to acclimate and adapt to the space environment. A multi-parameter facility would help unravel the effects of altered gravity in combination with other factors, either typical of space (i.e. ionizing radiation) or of confined volumes of cultivation chambers. Moreover, by modulating the exposure to specific environmental conditions (e.g., air flow or light), it would be possible to study the direct and indirect effect of the space factors on plants, bridging the knowledge gaps of the acclimation/adaptation mechanisms.

Currently, to study plant development in the space environment through the whole life cycle of a plant, including crops/food plants, is possible only using the ISS research platform: no other active platforms possess capabilities for answering the plant biology research questions listed in this paper. The infrastructure present on ISS can be modernized to achieve the needed goals to point to the sustainability of space exploration with BLSS.

Using the ISS platform for research will be the defining stepping stone into exploratory-class missions deeper into space. International efforts are ongoing to design and develop additional payloads in the frame of the Artemis program (e.g., NASA PRISM solicitation) (https://www.nasa.gov/feature/nasa-releases-prism-call-for-potential-lunar-surface-investigations). The Gateway platform is also currently planned for lunar orbit in the mid-late 2020 s. This should provide opportunities for experiments deeper into space, particularly outside of Earth’s magnetic field and the unique radiation environment can be exploited to further develop our understanding of the effect that different types of radiation may have on plants in combination with other space factors.

**Fig. 3:**
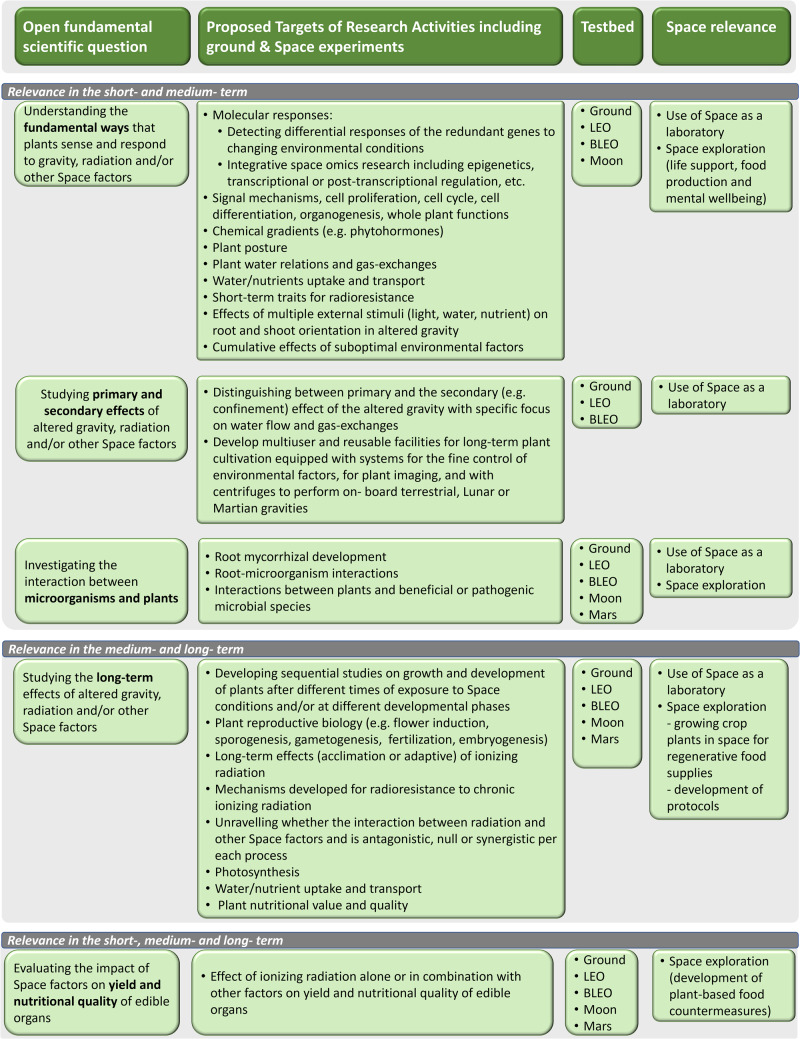
Summary of possible goals (targets) within the main knowledge gaps of plant biology research in space with priorities in the timeframe of 2022–2030 and beyond (short-term, 2022–2024; medium-term, 2024–2030; long-term, beyond 2030) using primarily the ISS platform. Other research platforms such as ground, Moon, Mars, LEO and BLEO (beyond LEO) are also included. They represent both the basis for the research on ISS and future research activities post-ISS (e.g. the GATEWAY orbiting the Moon).

It is not straightforward to indicate what are the more urgent goals of plant space biology since it has been recognized that space farming is becoming more and more a necessity as long as the roadmap for human space exploration goes beyond LEO (BLEO). A summary of possible goals (targets) within the main knowledge gaps (open fundamental scientific questions) identified in the previous paragraphs is reported in Fig. 3.

The first, second and third knowledge gaps should be addressed in experiments to be performed in the short- and medium-term since they will provide fundamental information that is the basis for the realization of more complex experiments in the long-term. The knowledge obtained in the short- and medium-term will be fundamental to defining requirements and developing new hardware to support long-term exploratory-class human missions. Indeed, in such missions, completely closed BLSS are themselves the “main requirement” although with technological differences depending on the scenarios determining the environmental constraints and mission duration.

In conclusion, to achieve successful space exploration, it is fundamental to apply an integrative approach in space biology merging together the information gained at different biological levels (e.g. molecular, cellular, tissue/organ up to the whole individual) as well as integrating knowledge among organisms (producers, consumers and degraders) also to understand the effect of space factors on their capability of networking in the artificial ecosystem as in nature on Earth. Space biology has been historically divided into sub-disciplines dealing with animals/mammals, microbes and plants without much interaction. However, some processes of altered metabolism as those regarding DNA-repair mechanisms as well as ROS and peptide signaling as stress responses are well conserved among species (including animals and plants). This paves the way towards the need for making synergies among disciplines to achieve an integrated picture of common *vs* distinct responses to space factors in different organisms. Possibly, the establishment of global standard operating procedures for space *omics* (including metagenomics) data sets generation and annotation, including but not limited to those generated within the framework of ESA and NASA projects, can allow the expansion of statistical power in space flight experiments by means of the federation of data sets.

Indeed, it has been recognized that to achieve BLSS operating in space and guarantee better protection of human health and well-being in space, an integrated, multidisciplinary approach linking together different branches of life science (e.g. animal and human physiology, plant biology, microbiology, etc.) is needed, as well as the cooperation with physical sciences, technologies and engineering. Therefore, there is a need to bridge and strengthen the interconnections between them also through the design, proposal, and realization of new studies and experiments.
